# Retraction Note: Extremely efficient aerogels of graphene oxide/graphene oxide nanoribbons/sodium alginate for uranium removal from wastewater solution

**DOI:** 10.1038/s41598-024-79455-3

**Published:** 2024-11-12

**Authors:** Ali A. Jabbar, Dhia H. Hussain, Kamal H. Latif, Salim Albukhaty, Adel Kareem Jasim, Ghassan M. Sulaiman, Mosleh M. Abomughaid

**Affiliations:** 1 College of Science/Chemistry Department, Mustansiriyah University, Baghdad, Iraq; 2 The Iraqi Authority for the Control of Radioactive Sources, Baghdad, Iraq; 3 Department of Chemistry, College of Science, University of Misan, Maysan, Iraq; 4 College of Medicine, University of Warith Al-Anbiyaa, Karbala, 56001 Iraq; 5 Division of Biotechnology, Department of Applied Sciences, University of Technology, Baghdad, 10066 Iraq; 6 Department of Medical Laboratory Sciences, College of Applied Medical Sciences, University of Bisha, 255, 67714 Bisha, Saudi Arabia

Retraction of: *Scientific Reports* 10.1038/s41598-024-52043-1, published online 13 January 2024

The Editors have retracted this Article.

After publication, concerns about unusual patterns in the XRD data presented in Figure 2 were brought to the attention of the Editors. Upon review, the Editors no longer have confidence in the reliability of the results and findings of this Article.

All Authors disagree with the retraction.

